# Magnitude and associated factors of depression among prisoners in Wollega zones, Oromia region, Ethiopia: A cross-sectional study

**DOI:** 10.1371/journal.pone.0260920

**Published:** 2022-03-31

**Authors:** Edosa Tadesse, Emiru Merdassa, Eba Abdisa, Tadesse Tolossa

**Affiliations:** 1 Wollega University Referral Hospital, Institute of Health Sciences, Wollega University, Nekemte, Oromia, Ethiopia; 2 Department of Public Health, Institute of Health Sciences, Wollega University, Nekemte, Oromia, Ethiopia; 3 School of Nursing and Midwifery, Institute of Health Sciences, Wollega University, Nekemte, Oromia, Ethiopia; Indian Institute of Technology Delhi, INDIA

## Abstract

**Background:**

Mental health is for everybody, but the individuals in prisons require more focus. Depression is a contributor to the global burden of disease and affects people in prisons in particular. There were limited studies on depression among prisoners. Therefore, the study aimed to assess the magnitude of depression and factors associated with it among prisoners in Wollega Zones Prisons, western Ethiopia.

**Methods:**

Institutional based cross-sectional study was conducted on randomly selected 368 prisoners from June 1, 2019, up to August 1, 2019, in Wollega zones prisons. Data were collected by trained data collectors through interviewer-administered questionnaires. Data were entered into EpiData version 3.1 and analyzed using SPSS version 20. Bivariable and Multivariable logistic regression model was computed to identify factors associated with depressive symptoms. In the final model, the strength of the association between independent variables and depression was measured using the Adjusted Odds Ratio (AOR) with the corresponding 95% Confidence Interval (CI). Then, in the final model, variables having a p-value of less than 0.05 were considered factors significantly associated with depression.

**Results:**

Of the total study respondents, 166/368 (45.1%) had symptoms of depression. Lack of job opportunity in prison (AOR = 6.64, 95%CI: 3.65, 12.06), not attending religious place at all (AOR = 3.51, 95%CI: 1.30,10.00), and Unsentenced for suspected crime (AOR = 7.36, 95%CI: 2.08, 26.04) were associated with depression.

**Conclusion and recommendation:**

This finding showed that the magnitude of depression in prisons was high. Prisoners in the young age group, attending religious places, being married, prisoners who were sentenced for suspected crime on timely and prisoners performing work in prison were less likely to have depression. The prison institution needs to facilitate income-generating activities in prison, promoting prisoners to attend their religious places and promoting timely sentencing.

## Background

Depression is a major depressive disorder characterized by at least two weeks of low mood, low self-esteem, loss of interest in normally enjoyable activities, loss of energy, disturbed sleep or appetite, feelings of tiredness, pain without a clear cause, and poor concentration [1, 2]. It affects all communities and imposes an enormous disease burden almost everywhere across the world and can cause suicide, loss of jobs and relationships, loss of productivity, and deterioration in physical health if it is not managed [3–5]. There were over 300 million cases of major depressive disorder worldwide and a significant contributor to the global burden of disease [6, 7].

An estimated 10 million people are in prisons worldwide of which the majority of them live in low- and middle-income countries and increased by one million per decade globally [8]. Compared to the individuals living in the community, prisoners were five to ten times more likely to develop depression [9]. Many studies reported different levels of depression among detainees, 46.1% among Norwegian [10], 59.4% among Central Prison of Peshawar, Pakistan [11], and 29% among in Iran [12]. Ethiopia’s prison population rate has increased from 94 to 124 per 100,000 of the total population between 2000 and 2011 [13, 14]. According to study conducted in different parts of Ethiopia, the magnitude of depression varies from prison to prison, 43.8% in Northwest of Amhara Regional State [15], 41.9% in Jimma town [16], 45.5% in Bahir Dar [17], and 56.4% in southern Ethiopia [18].

Several factors have a contribution to depression in prisons; overcrowding, various forms of violence, enforced solitude, lack of privacy, lack of meaningful activity, isolation from social networks, insecurity about future prospects like work and relationships, inadequate health services, lack of social support, dissatisfaction before and after imprisonment, gender, ages, the memory of past illegal acts and prolonged stay in the prison [19]. In Ethiopia, about 1.7% of the country’s health expenditure was expended on mental health services [20, 21]. Generally, the emphasis given to mental health was very low across the world in general and prisoners in particular. There has been some previous evidence for mental disorders in general and few studies have focused on depression in particular in Ethiopia [22–26]. There were limited studies conducted in Ethiopia, particularly in western parts to determine the magnitude of depression and its associated factors among prisoners. Therefore, this study was aimed to estimate the magnitude of depression and its associated factors with depression among prisoners in Wollega Zones, western Ethiopia.

## Methods

### Study setting and study period

The study was conducted in Wollega of Oromia, Ethiopia. Wollega has four zones; East Wollega zone, West Wollega Zone, Kellam Wollega, and Horo Guduru Wollega zone, and prisoners were randomly selected from each zone. Wollega zones are located on the west of Ethiopia and there were about 4845 inmates jailed [27]. The study was conducted from July 01, 2019, to August 30, 2019.

### Study design and population

Institution-based cross-sectional was employed. The source population for the study was all prisoners in Wollega Zones, while the study population was zone-level prisoners in four of Wollega Zones during the study period. Inclusion criteria all prisoners whose age were18 years and above were included in the Study. Prisoners with mental illness and severe medical illness who were unable to respond were excluded from the study.

### Sample size and sampling procedure

The sample size was calculated by using the single population proportion formula by considering the study conducted in 2018 in southern Ethiopia among prisoners that shows the prevalence of depression among prisoners is 56.4% [18] with assumptions of 95%CI, the margin of error 5%. The study population was 4845 which is less than 10,000 correction formula was used and considering a 5% non-response rate the final sample size was 368 prisoners. The sample was randomly selected from four zones of Wollega, 152 prisoners from East Wollega, 117 prisoners from West Wollega, 49 prisoners from Kellem Wollega, and 50 prisoners from Horo Guduru Wollega Zone based on proportion to their size.

### Data collection techniques and data quality assurance

Data were collected by face-to-face interviews with interviewer-administered questionnaires by a trained health professional working in a prisons clinic. Questionnaires were adopted from different literature reviews [11, 17, 18, 25, 28]. A structured questionnaire has five sections: a socio-demographic questionnaire to assess the resident’s background information. A questionnaire was translated into Afan Oromo and back-translated to English by translators to ensure consistency. The questionnaire was pre-tested on 5% of the total sample size of the study at the Buno Bedelle zone. Modifications concerning the clarification of the content and simplification of the wording were considered after the pre-testing of the questionnaire. Data were collected through a face-to-face interview. The collected data were checked for completeness, accuracy, clarity, and consistency on a daily basis by a supervisor at each facility. Any errors and incompleteness were corrected before the data analysis.

### Variable and outcome measurement

Patient Health Questionnaire (PHQ-9) which contained nine questions that measures a problem that the prisoners worried about in the last fifteen days were used to measure depression with scale measurements ranging from zero (not at all) to three (nearly every day) to assess an inmate’s symptoms of depression [29]. A participant who has a PHQ-9 score ≥ 5 was considered to be in a state of depression [18]. In addition, the Oslo 3-items social support scale was used to measure the strength of social support. The scores for the scale range from 3–14. The Oslo-3 scale has been used in several studies, confirming the feasibility and predictive validity with respect to psychological distress [30] and categorized into; poor support: prisoners who scored 3 to 8, moderate support: scored 9 to 11, and strong support: scored 12 to 14. The questionnaire assesses the prison-related factors, substance use such as khat, alcohol, and cigarette, the presence of chronic physical illnesses such as tuberculosis, cardiovascular diseases, HIV/AIDS, cancer, diabetes, asthma, and hypertension. Lifetime substance use was also defined as the use of at least one specific substance for non-medical use within their lifetime (alcohol, nicotine, khat, and cannabis use) before imprisonment was also assessed.

### Data management and analysis

Data were entered and cleaned using Epi-Info software version 7, and then exported to statistical package for social science (SPSS) software version 20 for analysis. Frequency and percentage were computed. Bivariable analysis was performed and independent variables who have a p-value < 0.25 were included in multivariable analysis [31]. The strength of association between the dependent variable and independent variables was expressed in odds ratio (OR) with a 95% confidence interval. Multivariable logistic regression analysis was used to obtain adjusted odds ratios (AOR) and determine the associations between the independent variables and depression. Statistical significance was set at p-value < 0.05. The multivariable results were interpreted with adjusted odds ratios (AOR) with 95% confidence intervals (95%CI).

### Ethical considerations

Ethical clearance was obtained from Wollega University Institute of Health Sciences and letters of co-operation were written to all administrative bodies of the prison and permission was secured at all levels. Written informed consent was obtained from the prisoners after a complete description of the purpose of the study. Privacy and confidentiality were also assured to each prisoner who participated in the study. Study respondents had the right not to participate in the study. Respondents diagnosed with depression were linked to the Psychiatry ward of their respective hospitals for the treatment.

## Results

### Socio-demographic characteristics of study participants

Three hundred and sixty-eight prisoners participated in this study and gave a response rate of 100%. The median age of the study participants was 21 years with an IQR of 7 and 44(12%) of the participants had never attended school. Three hundred twenty-two (87.5%) of the participants were Christian. The majority (83.4%) of the participants were Oromo and 53 (14.4%) of the participant were Amhara ethnic group. Regarding the duration of imprisonment 227(61.7%) of them were imprisoned for less than one-year, 134 (36.4%) of them were imprisoned for 1–5 years and 162 (46.7%) of the prisoners were sentenced to more than five years. Concerning the social support 192 (52.2%) of the prisoners had poor perceived social support and 13(3.5%) of the prisoners had good perceived social support (Table 1).

**Table 1:** Characteristics and percentage distribution of the of prisoners in Wollega zones of Oromia, Ethiopia, 2019.

Variable	Category	n	%
Age (Years)	18–24	243	66.0
	25–31	60	16.3
	32–38	16	4.3
	≥39	49	13.4
Gender	Male	353	95.9
	Female	15	4.1
Ethnicity	Oromo	307	83.4
	Amhara	53	14.4
	Gurage	8	2.2
Previous area of residence	Urban	106	28.8
	Rural	262	71.2
Marital status	Single	208	56.5
	Married	131	35.6
	Others	29	7.9
Educational level	No formal education	44	12.0
	Primary education	84	22.8
	Secondary or above	203	55.2
Religion	Muslim	46	12.5
	Christian	322	87.5
Previous occupation	Unemployed	281	76.4
	Employed	87	23.6
Perceived social support	Poor	192	52.2
	Moderate	163	44.3
	Strong	13	3.5
Duration of stay at prison	<1 year	227	61.7
	1–5 years	134	36.4
	>5 years	7	1.9
Sentenced	Yes	347	94.3
	No	21	5.7
Total sentenced in year	<1 year	60	17.3
	1–5 years	125	36
	>5 years	162	46.7

### Clinical and behavioral characteristics of the prisoners

Almost half, 182 (49.4%) of the participants had the habit of attending religious places for three or more days a week. Among study participants, 8(2.2%) of the prisoners had a family history of mental illness, 25 (6.8%) of prisoners were living with chronic diseases, 257(69.8%) of prisoners were lifetime Alcohol users (Table 2).

**Table 2:** Behavioural and clinical characteristics percentage distribution of the of prisoners in Wollega zones of Oromia, Ethiopia, 2019.

Characteristics	level	n	%
Living with chronic illness	Yes	25	6.8
	No	343	93.2
Life time Alcohol use	Yes	257	69.8
	No	111	30.2
Attending religious place	No	36	9.8
	Yes	332	90.2
Life time Smoking cigarette	Yes	67	18.2
	No	301	81.8
Life time khat use	Yes	93	25.3
	No	275	74.7

### Prevalence of depression

**Fig 1:**
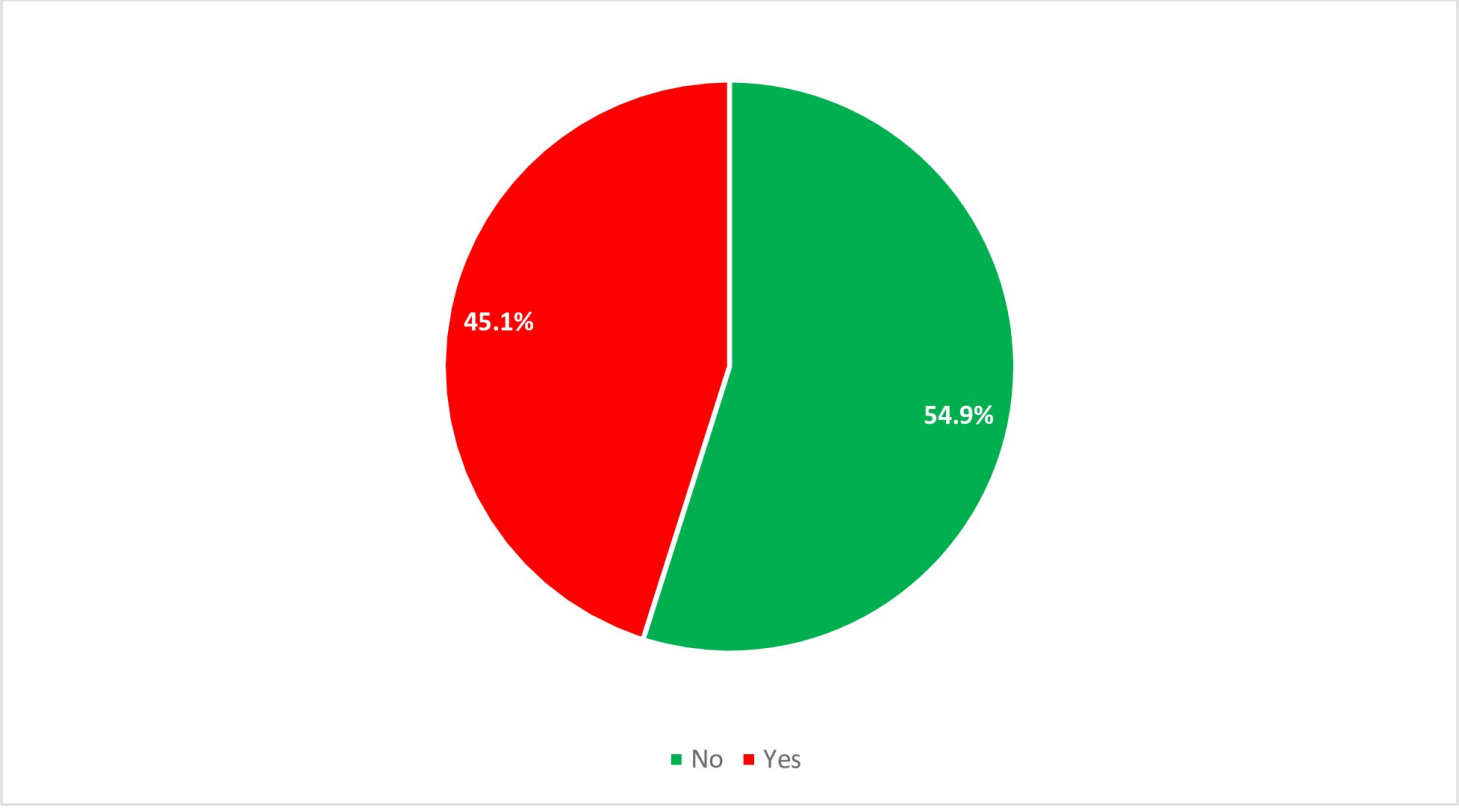
Proportiion of depression among prisoners included the study, Wollega zones, Western Ethiopia, 2019.

**Fig 2:**
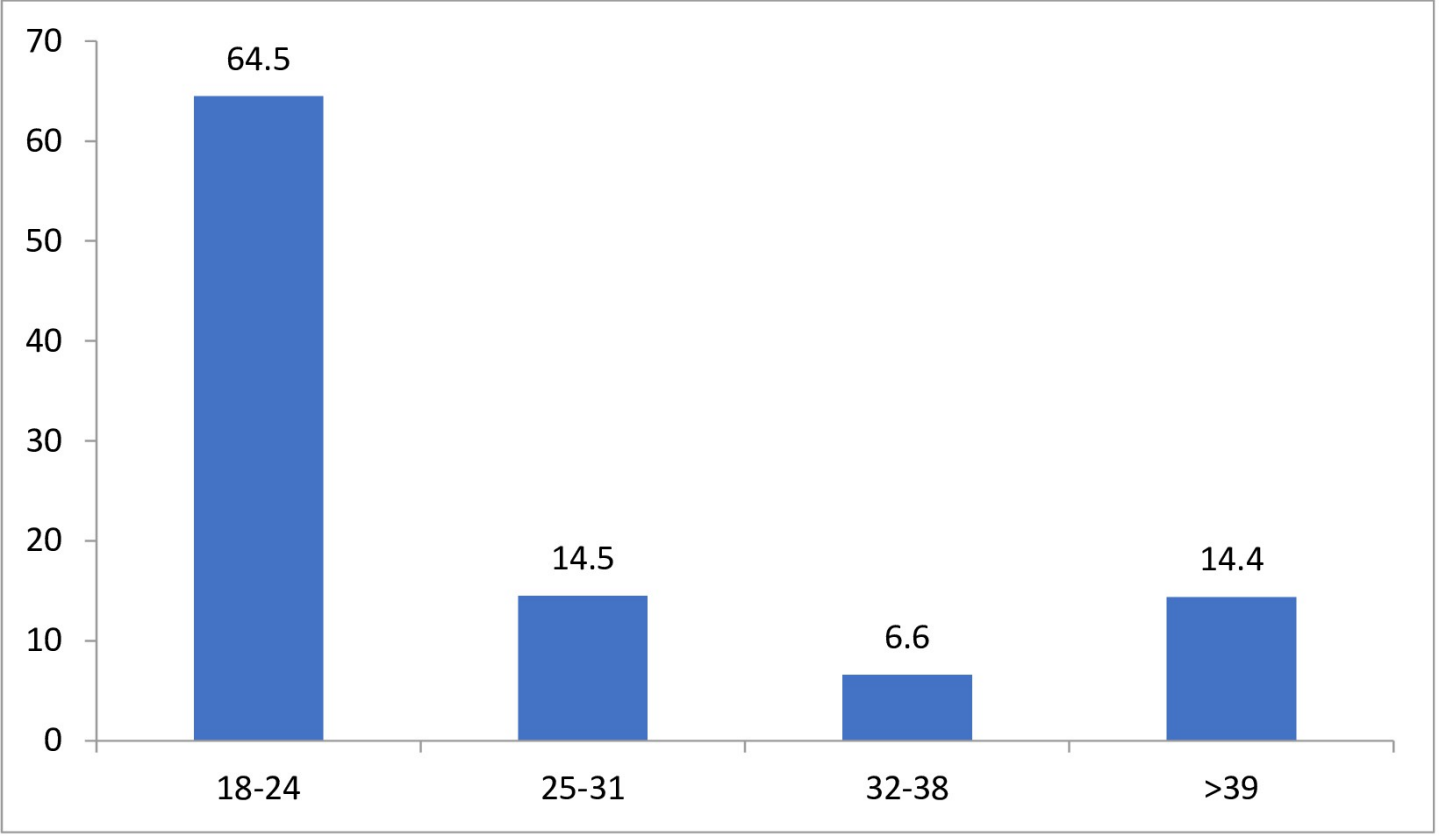
Graphical representation of magnitude of depression in by age group 2019, Wollega, Ethiopia.

Among study participants, 166 (45.1%) of study subjects were experienced depression (Fig 1). Nearly two-thirds, 107(64.5%) depressed prisoners were in the age group of 18–24 years (Fig 2).

### Factors associated with depression

Multivariable binary logistic regression analysis was employed. The finding showed that opportunity for a job in the prison, age, marital status, and getting sentence for the suspected crime had a significant association with depression.

The odds of having depression among prisoners who had no jobs in prison were 6.64 times more likely (AOR = 6.64; 95%CI: 3.65, 12.06) to develop depression compared to those who had the job opportunity in prison. Prisoners who were in the 18–24 age group (AOR = 0.16; 95%CI: 0.05–0.50) and 25-31years age group (AOR = 0.26; 95%CI: 0.09, 0.75) were less likely to develop depression compared with prisoners in the age group 39 years and above. Prisoners who were not got Sentenced for suspected crime were 7.36 times higher (AOR = 7.36; 95%CI: 2.08, 26.04) compared to those who got sentenced. Prisoners who were married (AOR = 0.28; 95%CI: 0.12–0.67) and Others (divorced, separated, and widowed) (AOR = 0.20; 95%CI: 0.05, 0.81) were less likely to develop depression compared to those who were single. Prisoners who were not attending religious places at all were 3.51 times (AOR = 3.51; 95%CI: 1.30, 10.00) more likely to develop depression than those who attend religious places in a week (Table 3).

**Table 3:** Factors associated with depression by multivariable logistic regression among prisoners in Wollega zones prisons, West Ethiopia, (n = 368).

Variable	Category	Depression		COR 95%CI	AOR 95%CI
		Yes (n, %)	No (n, %)		
Age	18–24	107(44%)	136(56%)	0.82(0.44, 1.52)	0.15(0.05, 0.50)
	25–31	24(40%)	36(60%)	0.69(0.32, 1.49)	0.26(0.09, 0.75)
	32–38	11(68.8%)	5(31.2%)	2.29(0.69, 7.58)	1.34(0.29, 6.15)
	>39	24(49%)	25(51%)	1	1
Availability of work in prison	Yes	30(24.2%)	94(75.8%)	1	1
	No	136(55.7%)	108(44.3%)	3.95 (2.44, 6.39)	6.64(3.65, 12.06)
Marital status	Single	102(49%)	106(51%)	1	1
	Married	51(38.9%)	80(61.1%)	0.66(0.43–1.003	0.29(0.12, 0.67)
	Others	13(44.8%)	16(55.2%)	0.84(0.39–1.84)	0.20(0.05, 0.81)
Sentenced	No	16(76.2%)	5(23.8%)	4.20(1.51, 11.73)	7.36(2.08, 26.04)
	Yes	150(43.2%)	197(56.8%)	1	1
Attending religious place	No	30(83.3%)	6(16.7%)	8.56(3.71, 19.72)	3.51(1.30, 10.00)
	Yes	136(41%)	196(59%)	1	1
Educational level	No education	4(9.1%)	40(90.9%)	1	1
	Primary	40(47.6%)	44(52.4%)	9.09(2.97, 27.68)	4.56(1.27, 16.31)
	Secondary & above	122(50.8%)	118(49.2%)	10.34(3.59, 29.80)	6.75(1.83, 24.90)
Previous occupation	Unemployed	172(61.2%)	109(38.8%)	0.33(0.20, 0.55)	0.93(0.42, 2.05)
	Employed	30(34.5%)	57(65.5%)	1	1
Social support	Poor	137(71.4%)	55(28.6%)	2.91(0.94, 9.04)	3.39(0.93, 12.41)
	Moderate	23(14.1%)	140(85.9%)	0.19(0.06, 0.62)	0.22(0.06, 1.81)
	Good	6(46.2%)	7(53.8%)	1	1

## Discussion

This study showed that the magnitude of depression was 45.1%, which is higher than the national estimate of depression in the community in Ethiopia [20] and a study conducted in southwest Ethiopia, Jimma [16]. Almost the same with the study conducted in Bahir Dar Prison, Ethiopia [17] and lower than the study done at Hawassa Central prison, Ethiopia [18]. Higher magnitude of depression in prison compared to the general population had been consistently reported from studies done in Nigeria [28], Brazil [23], Pakistan [11], and Iran [12] with some variations in magnitude among the studies. The difference in the magnitude of depression among the studies might be due to the variations in prison environments, characteristics of the study area, and sample variations that could be associated with the magnitude of depression.

This study also showed that prisoners who don’t have job opportunities were more likely to have depression compared with those who had to have job opportunities in prison. In support of this finding, the study conducted in Jimma Town Prison, South West Ethiopia [16] and a study conducted in Hawassa prisoners, SNNPR, Ethiopia [18] had reported prisoners who have participated income-generating activities were less likely to have depression. This might help prisoners more adhere and focused on their works and activities which might contribute to reducing depression. Young age group prisoners were less likely to develop depression compared to the prisoners in the age 39 and above. A study conducted in Jimma Town Prison, south West Ethiopia had reported contrary to this finding [16]. This possible explanation for this difference might be socio-demographic characteristics of the study participant, variations in prison environments, sample size, and variations in the overall characteristics of the study setting.

Prisoners who were not attending religious places at all were more likely to develop depression than prisoners who attend religious places. This study is in line with the study conducted in Malaysian [19]. Religious practice may help to cope better with the stressful life and hope in life which creates a supportive environment, lessen risk and prevent depression.

Prisoners who were not sentenced yet for suspected crime were more likely to have depression compared to those who got sentenced. This study is similar to the study conducted in Malaysian [32]. Pending court cases might be a stressful period and prisoners are vulnerable to extra stress from uncertainty and proximity of possible sentences which might precipitate depression.

Prisoners who were in the married marital status group were less likely to have depression compared to those who were single. This is contrary to the study conducted in Ethiopia concerning the magnitude of depression and associated factors in Ethiopia: findings from the National Health Survey [20]. This possible explanation for this difference might be socio-demographic characteristics of the study participant, variations in prison environments, sample size, and variations in the overall characteristics of the study settings. This study used the standardized screening tool with high reliability to screen depression regardless of population characteristics. This has some limitations, the study was institution based which could limit its generalizability to normal population and clinical setting, and also, in this study a cross-sectional design was used and the data were also prone to recall bias.

### Conclusions and recommendations

This finding showed that the magnitude of depression in prisons was high in the study setting. Prisoners in the young age group, attending religious places, being married, prisoners who were sentenced for suspected crime on timely and prisoners performing work in prison were less likely to have depression. The institution needs to facilitate income-generating activities in prison, promoting prisoners to attend their religious places and promoting timely sentencing.

## Supporting information

S1 Dataset (SAV)

## Abbreviations

AOR: Adjusted Odds Ratio
CI: Confidence Interval
COR: Crude Odds Ratio
GBD: Global Burden of Disease
E.C: Ethiopian Calendar
ETB: Ethiopian Birr
MDD: Major Depressive Disorder
PHQ-9: Patient Health Questionnaire-Nine
SNNPR: Southern Nation and Nationality peoples Region
OR: Odds Ratio
SPSS: Statistical package of social science
WHO: World Health Organization
